# Family planning integration in Ethiopia’s primary health care system: a qualitative study on opportunities, challenges and best practices

**DOI:** 10.1186/s12978-023-01709-6

**Published:** 2023-12-01

**Authors:** Addisalem Titiyos, Yohannes Mehretie, Yibeltal Kiflie Alemayehu, Yohannes Ejigu, Kiddus Yitbarek, Zelalem Abraham, Kathryn A. O’Connell, Jemal Kassaw

**Affiliations:** 1 EngenderHealth, Addis Ababa, Ethiopia; 2 Jimma University, Jimma, Ethiopia; 3 Addis Ababa, Ethiopia; 4 EngenderHealth, Washington, DC USA

**Keywords:** Family planning, Contraceptive, Integration, Primary health care, Family planning integration, Ethiopia

## Abstract

**Background:**

Family planning (FP) service integration into primary health care (PHC) is an effective approach to realize reproductive autonomy, increase the use of contraceptives, and improve maternal and child health outcomes. The Ethiopian government promotes integration of FP services into primary health care (PHC). However, there is paucity of evidence on the status of FP service integration. The aim of this study is to explore the state of FP integration into PHC services and identify facilitators and barriers to integration.

**Methods:**

A qualitative study nested with a larger national study was conducted from July to October 2022. A total of 60 interviews were conducted with FP stakeholders including, government organizations, non-governmental organizations, donors, service providers, and clients. Interviews were audio recorded, transcribed, and coded using OpenCode 4.03. The coded data were analyzed using framework analysis approach, using the Primary Health Care Performance Initiative (PHCPI) framework. Direct quotes and results from the coding and categorization were used to develop the report.

**Results:**

Family planning is largely provided in designated units by dedicated staff within PHC facilities. The provision of integrated FP service within each service unit is in its early stage. Successful examples of integration include integration of FP with postnatal care, abortion care, and youth-friendly service centers. Facilitators of integration include commitment of the government and partners, the presence of policies and guidelines, and positive attitude of service providers and clients. However, integration of FP also faces challenges that are largely related to challenges of the FP program even before integration. These include resource shortage, health workers shortage, health workers’ capacity/skill gaps, misconceptions about FP, religious and socio-cultural norms, and lack of awareness.

**Conclusions:**

Integration of FP with PHC services in the Ethiopian public health facilities is viable. Pre-existing challenges of the FP program continued to be barriers to integration. Expanding the experiences of good practices in the integration of FP with post abortion care, post-natal care, and youth-friendly service centers to other components of PHC warrants attention. Addressing both supply- and demand-side challenges of the FP program is needed to facilitate the integration of FP with other PHC services.

## Plain language summary

This study aimed to explore the status of integrating family planning (FP) services into primary health care (PHC) in Ethiopia and identify the facilitators and barriers to integration. The study included 60 interviews with stakeholders, including government and non-government organizations, service providers, and clients. We found that while dedicated FP staff provide services in designated units within PHC facilities, the provision of integrated FP services within other PHC units is in its early stage. Integration with post-abortion care, post-natal care, and youth-friendly service centers showed exemplary results that need to be used as good practices. The study identified challenges including resource shortage, health worker shortage, misconceptions about FP, religious and socio-cultural norms, and lack of awareness. The positive attitude of service providers and the commitment of the government and partners were identified as facilitators of integration. The study recommends expanding the successful practices of FP integration to other components of PHC and addressing supply- and demand-side challenges to facilitate the integration of FP services with PHC.

## Background

Access to comprehensive family planning (FP) and reproductive health services is an important determinant of women’s ability to exercise their reproductive autonomy and a key facilitator of gender equality and women empowerment [1, 2].It is also recognized as one of the most important life-saving interventions for women and children [3]. As a result, FP and the broader reproductive health has been a global priority for decades, even at times of emergencies [4–7]. However, unmet need for FP is still a major challenge in developing countries, despite decades of efforts to increase access to and utilization of FP [8]. An estimated 164 million women in the world have unmet need for FP in 2021, leading to unintended pregnancy, abortion and maternal and child mortality [9]. Satisfying unmet need is believed to prevent unintended pregnancies, unsafe abortions, and improve maternal and child health [10, 11]. In Ethiopia, although contraceptive use has been improving over the last two decades because of multifaceted intervention such as improving FP access in health facilities, building health care providers capacity, developing, and reviewing FP interventions guidelines and strategies, and improving FP commodities and supply management among others, the unmet need for FP among Ethiopian women is still very high [12]. In 2016, 22% of married women had an unmet need for FP [13].

Integration of FP services into primary health care (PHC) is an effective approach to improve FP use and maternal and child health outcomes [14]. A number of studies have shown that integrating FP into maternal and child health (MCH) services increases access to and the use of modern contraceptives, improves efficiency and decreases missed opportunities, reduces unintended pregnancies, and improves maternal and child health outcomes [15–19]. Recognizing the available evidence, the World Health Organization (WHO) recommended to integrate FP, specifically during postpartum period with MCH services [16]. However, there are also concerns that integration could over-burden health care workers, leading to burnout and reducing the quality of the care [14].

A few studies from Ethiopia also assessed FP integration with MCH services and reported on its effectiveness. Studies on the effect of FP integration with HIV care services reported improvement in FP uptake and declines in unmet need for FP among women of reproductive age group with HIV infection [20, 21]. A longitudinal study also demonstrated that FP counselling during postpartum and during child immunization period were associated with increased modern contraceptive uptake [22]. However, integration of FP with other PHC services is still not strong [23].

In 2021, the Ethiopian health sector adopted a national guideline to strengthen the level of FP integration with PHC services. The guideline recommended integration of FP with a wide range of PHC services including abortion care, maternal health services (antenatal, delivery and postnatal care), child care services including immunization, HIV counseling, testing and care services, inpatient and outpatient services, and the model of integration can be either using an internal referral mechanism or direct provision of FP services depending on the context [24]. Understanding the facilitators and barriers of integration is critical to ensure successful implementation of the guideline and make necessary adjustments in strategies as needed. However, evidence on the facilitators and barriers to integration of FP in the PHC system of Ethiopia is scanty and focus only on a few specific services.

To inform the government’s efforts of strengthening the FP services through integration, EngenderHealth conducted a comprehensive study collecting quantitative and qualitative data from diverse stakeholders, to generate evidence on different aspects of FP integration into the PHC system. This article reports the perspectives of health managers in the public sector, non-government partners, service providers, and communities on the status, opportunities, challenges, and best practices in the effort to integrate FP with PHC services.

## Methods

### Analysis framework

**Fig. 1:**
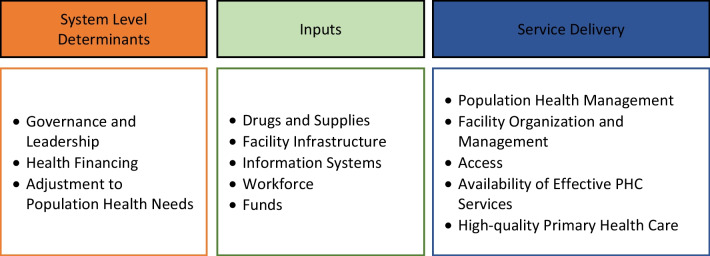
PHCPI Domains selected for assessing integration of family planning with PHC system in Ethiopia

We adapted the Primary Health Care Performance Initiative (PHCPI) framework to guide our analysis. The PHCPI framework includes five domains including system level determinants, inputs, service delivery, outputs, and outcomes that include 13 sub-domains constituting the critical components of a strong PHC system [25]. In this study, we focused on integration of FP across the first three of the five domains in the PHCIP framework, including system level determinants, inputs, and service delivery (Fig. 1).

### Study area and setting

Ethiopia is a low-income country located in the horn of Africa. In 2022, the population of Ethiopia was estimated at 105.2 million of which 77.3% live in rural areas [26]. The country is divided into 11 administrative regions and two city administrations. The health system of the country has three tiers, namely, primary, secondary, and tertiary. Primary health care units (composed of health centers and health posts) represent the most accessible part of the health service delivery system and are responsible for the provision of the primary level care. Family planning is one of the components of PHC delivered primarily at the levels of health posts and health centers [27].

### Study design

We conducted a qualitative study following a phenomenological approach nested under a larger national study conducted from July to October 2022. We collected qualitative data using key informant interviews with key FP stakeholders working in governmental organizations, non-governmental organizations and donor agencies focusing on their real experiences in implementing FP with the Ethiopian PHC system.

### Study participants

The study collected data from all categories of actors at all levels of the health system. We selected officials and/or experts for interview through a purposive sampling strategy aiming to identify informants with rich experience and expertise. A total of 60 interviewees, representing health institutions at different levels of the health system, experts from non-government partner organizations, and FP clients participated in the study. These include policy makers and program managers at the federal and regional levels (n = 7); woreda health office and health facility managers (n = 18); health service providers (n = 12); FP clients (n = 11), and representatives of non-government partner organizations and other stakeholders (n = 12).

### Inclusion and exclusion criteria

The study included only public-owned health facilities, and health facilities in conflict areas were excluded.

### Data collection procedures

We reviewed relevant literature, discussed with stakeholders and among the research team of EngenderHealth in the development of data collection guides. We used semi-structured interview guides to collect qualitative data through key informant interviews with experts and in-depth interviews with clients. Seven experienced qualitative research assistants collected qualitative data after receiving a three-days training by the research team. The training included the study objectives, methods of data collection, and data collection guides. Key informants were informed about the study purpose and their role in it a day before data collection days. Appointments were scheduled for the following day at a time convenient for them and interviews were conducted in their respective offices. The data collectors conducted a large majority of the interviews through face-to-face interviews; however, some of the key informants, who were not available during the period of the data collection, were communicated via emails and interviewed using virtual platforms. The duration of the interview was 45-60 mins with each key informants and the interviewees were not reimbursed for their time.

### Data compilation, processing, and analysis

The data collection research assistants recorded all data collection activities and transcribed them verbatim in the languages of interviews. Then, we translated the verbatim transcripts into English and imported them to OpenCode 4.03 [28] for coding and categorization. We coded the data using two coding frameworks predominantly through a deductive approach. The first coding framework included the domains and sub-domains of the PHCPI framework, including system level determinants, inputs, and service delivery. The second coding framework reflected the study objectives, and it allowed indexing the dataset based on the relevance of each segment of text data to the key research questions, including status of integration, challenges of integration, opportunities for integration, best practices, and recommendations. The research team discussed and agreed on the definitions of each code prior to the start of coding. Then one of the research team members coded the data using the agreed upon coding framework. A new code—relevance of integration emerged during coding, and it was added in the second coding framework.

We generated code reports for synthesis and report writing. The research team read and reread each code report and synthesized the key findings under thematic areas that are produced by refining and merging codes in the initial coding frameworks. We used framework analysis approach to analyze the coded and categorized data. We also used direct verbatim and results from the coding and categorization to develop a report. The findings are finally presented in narrations supported by anonymized quotes as needed.

### Ethical considerations

We obtained ethical clearance from the Research Ethics Committee of the School of Public Health and College of Health Sciences at Addis Ababa University (Ref: 031/22/SPH) and written permissions to perform the study from the respective Regional Health Bureaus and offices of the sample woredas (administrative division equivalent to districts). All data collection instruments included informed written consent forms in which the consent of the respondents was ensured. The research team applied standard procedures to guaranteeing confidentiality during data collection fieldwork, analysis, and storage.

## Results

### Overview of findings

**Fig. 2:**
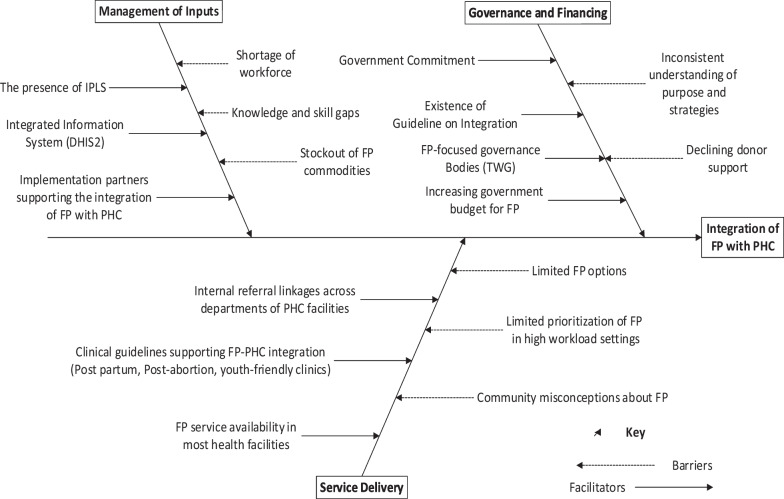
Summary of facilitators and challenges of FP-PHC integration

The results section of this article is organized under six main themes. The themes and sub-themes were the results of a combination of the PHCPI domains with the key concepts related to the study objectives; the main themes include the current status of integration, challenges, opportunities, good practices and recommendations forwarded by respondents for the future improvement of the program. Findings under each of these themes are presented under sub-sections on the integration of service delivery, management of inputs, and governance and financing. One additional thematic area emerged during coding—Relevance of FP integration with PHC. Findings on this thematic area are presented as a separate sub-section. Figure 2 presents a summary of the facilitators and barriers of FP integration with PHC.

### The relevance of integration of family planning with PHC

Key informants reflected on the relevance of FP integration with other PHC services. Managers and health care workers at all levels of the health care system (including MOH, regions, Woredas, and health facility managers) highlighted that integration of FP with PHC is appropriate for the Ethiopian context. In their interviews, key informants mostly linked FP—PHC integration with service delivery outcomes such as minimized missed opportunities for FP services, improved privacy and confidentiality for young clients, and more efficiency in terms of saving time for clients. For instance, a FP case team member from the MOH explained the relevance of FP integration with PHC and the efforts by the MOH to realize full integration as follows:

“...there is 70% unmet need for post-partum FP (PPFP), and efforts are made to increase the PPFP uptake through integration. The other strategy is integration of FP with abortion service. The ministry is also working to increase service uptake as well. A guideline is developed on the FP service integration to address the missed opportunities such as mothers who come to the facility for other services such as EPI, under 5 clinics, ART clinics, ANC and PP.” Key informant, FP case team member, MOH.

A health facility manager from a hospital in Oromia made a recommendation that family planning should be integrated with all hospital services.

“It is important to ensure that everyone who comes to this hospital has an understanding of family planning. This should include people who may come to our hospital for various reasons … sick or to be treated, … not only for women who are ready to give birth but for all women.” Key informant, health facility manager

Among interviewees, there were no objection against the concept of integrating FP with other PHC services.

### The current state of family planning integration with PHC

#### Service delivery

Family planning services are provided in all PHC facilities, including health posts, health centers, and primary hospitals. Most hospitals and health centers provide both short acting contraceptives, including combined oral contraceptives, injectable contraceptives, IUCD, and Implants. In addition, hospitals provide surgical contraceptives, primarily bilateral tubal ligation. Health posts are also expected to provide a range of methods, including long-acting FP, but in some of the health posts, long-acting FP service was not available, at least temporarily, during the period of data collection.

Family planning is largely provided as a standalone service in a designated unit and by dedicated staff instead of as part of services integrated within each service unit. Key informants reported that there are efforts to integrate FP with abortion care, postpartum care, and HIV care; however, the level of integration with other PHC services is sub-optimal. Describing the state of FP integration with PHC, the MCH director at the MOH said the following:

“... FP has already been integrated with abortion services as it is one component of the abortion service package. However, PPFP service is one of the programs which hasn’t been well implemented although it has a high demand. Currently 9% of health facilities with high client loads are giving PPFP...... Supported by partners, FP is also integrated with immunization and HIV care services in a few health facilities … this has a huge impact in addressing the mothers’ needs. MOH is working to scale up the integration efforts mainly with MCH and other service areas.” Key informant, Director of MCH Directorate, MOH

Some key informants reported that health facilities that are supported by development partners provide FP services either through referral or in one room by the same provider.

“Health facilities which had support from partners (training) are providing integrated services through internal referral or in the same service unit. For example, FP service training is included in comprehensive abortion care training thus the service is integrated at the service delivery point. Furthermore, the service is integrated with ANC, EPI and PP, where the same provider gives the service. In other facilities, FP service integration is implemented through internal referral after the mother gets adequate information.” Key informant, Packard Foundation

There is no uniformity in the conceptualization of integration of FP with PHC. For some of the key informants, integration means providing family planning counseling and linking women with the family planning unit through referral while for others, integration means delivering the full range of family planning services (counseling as well as methods). A manager from a primary hospital for example argued that FP is already integrated with PHC in his hospital but at the same time mentioning that the service is provided in a unit dedicated for FP and staffed with experts assigned specifically for FP.

“All types of services are provided in the institution. As I mentioned earlier, it has its own section; it has its own experts. It is integrated … I mean it has its own expert … it is provided independently during all shifts.” Key informant, Health Facility Manager

In health facilities where integration of family planning with other PHC services is aspired through a standalone family planning unit, information and counseling on FP is provided by health professionals in other units and willing clients are linked to the FP unit through internal referral. A facility manager from a health center in Amhara region described this arrangement by saying:

“Our health center has a separated FP unit. If mothers come for family planning services, we link them with the FP unit directly. When mothers come to OPD for other services, we counsel them about family planning. If they are willing to receive FP, we have a referral system to link them with the FP unit.” Key informant, Health Facility Manager

Another health facility manger from a health center in SNNP described a similar arrangement in which FP counseling has been integrated with different PHC service delivery platforms.

“EPI and PNC service areas are best sites to find eligible mothers. In immunization and ANC rooms, mothers always receive counseling on FP. During the last ANC visits, we create awareness among mothers on FP. We also counsel mothers who come for immunization services and link them with the FP unit … when mothers come to the OPD for other services, we link them to the FP unit ... In addition, there is screening of mothers who need nutritional support for their children. When mothers come to this program, we provide health education.” Key informant, Health Care Provider

Another manager from Aleta Wondo argued that linkage through internal referral is one form of integration. The informant said:

“Generally speaking, the arrangement is integrated; all the departments feed each other and are linked to the FP unit. At health center level we provide the service as an integrated program.” Key informant, Head of WoHO

In some of the study health facilities, clients have witnessed the integration of FP counseling services with other PHC services. A mother who visited a primary hospital to vaccinate her child reported:

“Today I came here to get immunization for my child. The health providers that immunized my newborn asked me about FP methods and advised me to get FP methods in that department. She (the health care provider) has informed me about the benefit of using FP method for me and my child, so, Inshallah, I will come and get FP method.” Key informant, Mother attending EPI Unit

Provision of the full range of family planning services, including both counseling and method provision, were reported for selected services. These include delivery and postnatal care, abortion and post abortion services, and youth-friendly service centers. A health facility manger described this type of arrangement by saying:

“Mothers who receive PAC services receive on-site family planning counseling services. If they are willing to receive family planning, they will receive FP services at the same area where they have received PAC … by the same provider” Key informant, Health Facility Manager

#### Management of inputs

Family planning supplies are provided to service delivery facilities through the health sector’s supply agency—EPSA, along with other drugs and medical supplies. There is no parallel supply chain system for FP commodities. As discussed by a respondent, FP commodities are provided to the health facilities regularly in a better way compared to other drugs and supplies.

“…there is not much problem with MCH supplies, resources are coming regularly, especially these FP kits are provided in complete form.” Key informant, EPSA

There is a partially integrated information system. At the level of recording and reporting, FP registers are already integrated with some PHC services, including postnatal care and abortion and post-abortion care. However, other service registers available at the PHC level do not have spaces for recording FP services. Integration is at its highest level for reporting. The DHIS2 tool provides a platform for reporting all PHC services in a single service delivery reporting system.

#### Governance and financing

Overall, there is high level of commitment to integrate family planning with other PHC services at different levels of the health system. The national policy documents support the integration of family planning services with other PHC services as FP is the government’s priority. There is a political commitment and ownership especially at the ministry of health level. The development of integration guideline, inclusion of integration data elements on the DHIS2 system and inclusion of FP on the IPLS were mentioned as enabling factors. The adoption of the integration guideline is considered as a key milestone for the realization of integration.

Family planning-focused governance structures include the FP technical working group at the national level, which is composed of different stakeholders from the Ministry of Health and other partner organizations.

“There is favorable environment for FP program in Ethiopia … this includes the adoption of the national FP guideline with the involvement of different stakeholders, the high level of commitment from MOH and RHBs, and the participation of civil society organizations in the planning and implementation of the program.” Key informant, CORHA.

According to a key informant from CORHA, integration of FP with PHC is possible in Ethiopia, but it depends on the commitment of the leadership and prioritization of competing problems. As a result, the current level of integration varies from region to region with regions affected by the recent conflict having the lowest level of integration.

“PHC facilities are ready to provide integrated FP service but there are regional disparities because of competing priority agendas such as reconstruction of distracted health facilities and unavailability of supplies and health professionals due to ongoing conflicts. In these areas, service uptake is also reduced as it [family planning] is perceived as a luxury in the context of conflict … In other areas, the FP service integration depends on the leadership and commitment of health facilities. Inconsistency in the attention given to the program due to high turnover among the leadership is a source of disparities in the level of integration of FP services with PHC.” Key informant, CORHA.

However, according to some key informants, there is no uniformity in the understanding of health managers regarding the desired levels of integration, this ranged from a commitment to moving towards full integration of FP counseling and method provision with all PHC services to consideration of integration as a strategy to cope up with shortage of space for a standalone family planning unit. These variations have resulted in inconsistencies in advocacy efforts.

### Challenges

#### Service delivery

Key informants repeatedly mentioned that the challenges to integrate FP are associated with shortage of resources, including health worker shortage and capacity or skill gaps. Health posts constitute a major component of the health service delivery model for the provision of PHC in Ethiopia. Integrating FP services at the health post level has worked very well for short-term methods. However, providing a full range of method mix at this level is a challenge for several reasons, including sub-optimal skills of HEWs, inadequate space, and shortage of medical equipment.

Explaining the challenges of FP integration with PHC, a key informant from UNFPA said the following:

“The challenges for FP integration can be categorized in to four: first, inconsistent advocacy and implementation of the initiatives at the national level; second, supply shortage; third, service provider related challenges such as lack of promotion and counseling of the FP service to clients who came for delivery and abortion service, because of lack of awareness of the providers; fourth, client’s refusal regardless of the counselling.” Key informant, UNFPA

On the other hand, from the demand side, there are a lot of misconceptions in the communities even though there are improvements these days, according to most of the respondents. Challenges such as lack of awareness in a pastoralist area; misconceptions related to the side-effects like infertility, delayed pregnancy, and contraceptives causing other health problems; religious and socio-cultural norms are mentioned as demand side barriers.

A key informant working in a pastoralist setting discussed that community perception towards using contractive methods.

“In the culture of Afar, it is condemned to use contraceptives especially in rural areas. … The community believes that, if they have more children there will be wealth and children are considered as a sign of honor”. Key informant, implementing partner.

Key informants stated that women from pastoralist settings feel that using contraception is shameful since their level of awareness about FP is very low. In agrarian settings, however, cultural issues are not significant problems for the use of FP service even though there is lack of awareness and inappropriate use of the services. Another discussant from agrarian area pointed out a common misconception among mothers in relation to the need for FP as:

“Mothers are usually reluctant to use FP services, and they are saying that ‘pregnancy cannot occur within 1 or 2 years after giving birth’.” Key informant, WoHO.

A health care provider discussed the challenge related to societal belief and perception in delivering FP services, even for a woman in need of the service.

“The tradition affects the FP service. First, they [women] are not allowed to use FP due to cultural and religious beliefs, which decrease FP usage. … Furthermore, when they pass all these challenges and come for services, they may not get the FP methods they want to use. Because they should hide it from their husband. For example, she may want Implanon [Implant contraceptive] but to hide she should use injectables instead”. Key informant, Health Care Provider

A couple of key informant panelists discussed the challenge with FP commodities’ supply chain related bottlenecks the affected delivery of service and FP integration in to the PHC setting. For instance, a respondent from Ethiopian Pharmaceutical Supply Agency (EPSA) hub discussed a gap in completing request and refill forms by health facilities and trained staff turnover.

“We respond to the health facilities’ needs based on their request for FP products. Others we cannot do anything if the facility fails to ask. We believe there are items that should exist in every facility and if we find some facilities that do not have such items, we are forced to ask in such cases why they didn't request these items through phone.” Key informant, EPSA hub focal person

The respondent also added:

“There is a high turnover in the pharmacy department of several health facilities. Those who have good skills and training are leaving their health facilities. This situation sometimes creates miscommunication.” Key informant, EPSA hub focal person

On the other side, a challenge in getting required commodities/supplies in a timely manner was discussed by another key informant respondent.

“The EPSA hub is supposed to provide supplies based on our report. They usually have a shortage of medical supplies on their hand and this in return negatively affects the amount of medical supplies they give to us. The other is the issue of late reporting to EPSA which delays the delivery of these consumable supplies.” Key informant, WoHO

According to one of the key informant discussants, poor data recording and reporting was discussed as another major challenge.

“Due to shortage of the necessary tools, we had problems with data accuracy, quality and timeliness. If these need to improve we must get the tools to enable us to integrate our data with health information service.” Key informant, WoHO

Situations affecting the mobility of people from place to place, transportation of medical supplies and commodities, discussed as a challenge to seek and provide services.

“To tell you the truth I used to stay at home during that initial COVID-19 surge, and everybody afraid to go to the hospital and even there were times when the city was locked, and I didn't come to the hospital at the time even if I become sick because of getting COVID-19. I also heard that, there were many women who got pregnant during COVID-19.” In-depth interviewee, Client

“…we have been facing security issue around Metekel area some time ago, in fact, the problems are still there. In this situation what we are doing is, we transport commodities to Chagni and they [health workers] will come and take it accompanying with ambulance.” Key informant, Health care provider

According to most of the respondents, there is a shortage of competent, sufficient and motivated human resources. Trained personnel are not available in most of the remote health facilities. According to the respondents, integration of FP services has brought additional tasks in the health facilities that need to be undertaken by additional health professionals. However, the reverse is happening, where there is high turnover. This made a few health professionals overburdened. On the other hand, the respondents repeatedly mentioned the skill and knowledge gap among health professionals because they did not take specific training for the service.

Further, the interviewees also reported the external incidents such as the pandemic of COVID 19 have affected FP service delivery and uptake at the PHC level. Especially in the Ethiopian fiscal year of 2012 (2020 GC) and 2013 (2021 GC), the number of FP service users has decreased remarkably. Nevertheless, since 2014 Ethiopian fiscal year (2022 GC) the FP service uptake has started increasing.

#### Management of inputs

Family planning service integration requires the availability of health workers’ training on FP service provision. Not all health professionals providing PHC services are trained in FP counseling and service provision. A shortage of skilled health professionals is a challenge particularly for the provision of long-term contraceptive methods.

“We have a good number of workforces to provide the service, but they need special training to provide the long-term FP services. Currently we have only two experts who can provide long-term FP services. Sometimes these professionals might be off duty due to different reasons, and when that happens there will be gap in the provision of the service.” Key informant, WoHO

In health facilities with high workload for PHC services, shortage of health professionals is a reason that deters the integrated delivery of FP with other PHC services. A health facility manager from a hospital in Oromia region for example reported that postpartum family planning is deprioritized by health professionals when they are overburdened with large numbers of laboring mothers.

“The challenge we face in this work is that we have a shortage of professionals for counseling. For example, women do not get enough counseling after childbirth because for example more than 20 women go into labor in one night, and only three people provide services; they [the health professionals] focus on early discharge of mothers to their homes without providing adequate counseling [family planning counseling] in the maternity ward.” Key informant, health facility manager from a hospital

The integration of the supply chain system for FP commodities with EPSA through the IPLS has led to longer lead time and higher frequencies of stockouts and overstocks. Stockout of FP commodities is a challenge for some health facilities. Preferred methods tend to be out of stock more often. That is believed to push clients away. A FP client from a health center described the consequences of stock out of injectable contraceptives as:

*“… As I have said earlier there is shortage of depo in the hospital this has resulted the mother to refrain from using FP because depo is their preferred contraceptive.” Key informant, Family planning client, Health Center*

#### Governance and financing

Although there is sufficient policy support to implement the integrated FP services at the PHC level, shortage of resources dragged the performance backward. The challenges on the national program as mentioned by the respondents are, gaps in the implementation of policies and guidelines, declining of funds by donors and gaps such as poor forecasting and procurement on the supply chain. According to key informants, there is an increasing trend in government budget allocation. However, it is not sufficient to overcome the challenges related to declining donor resources and increasing procurement costs for FP. A key informant explained the challenges related to shortage of finance for FP as follows:

“Although the budget allocated from the government has increased over time, donors’ fund has declined, whereas procurement related expenses and demand has increased, which has led to shortage of finance.” Key informant, CHORA

The ownership and leadership of the FP program witnessed at the ministry of health declines at regional, zonal and woreda levels. Consequently, there is less focus, lack of accountability, misconceptions and limitations related to planning and budgeting for the FP program. Furthermore, the high turnover of trained leaders and health care providers at the PHC level imposes a challenge on the program implementation.

“Even though there is a national interest and guideline has been developed, the lower level implementation is at infancy stage. The challenges include lack of attention from program coordinators due to competing agendas.” Key informant, Packard Foundation.

### Opportunities

#### Service delivery

The presence of PHC services that are more widely accepted by communities than FP present an opportunity for increasing access to FP services. In places where FP has been integrated with other PHC services, counseling on FP is provided and services are offered for clients who come for other PHC services. This type of arrangement was recognized as important particularly for women who are under the influences of anti-FP attitudes of family members (partners) and religious leaders by creating an opportunity for a more confidential use of FP services. A health facility manager explained this occasion as follows:

"Availability of several services like ANC, delivery, postnatal and EPI give the chance to meet the target audience that have difficulty due to the religious leaders and husbands that protect their wives not to use FP methods to secretly use FP if the service is integrated with other services; it gives them opportunity and confidence to use the methods at any time.” Key informant, Health Facility Manager.

By creating the opportunity for providing more confidential family planning services, integration can address one of the major barriers to the use of family planning methods, decrease missed opportunities for providing FP services, and increase service uptake. In facilities where health workers are trying to integrate services, women are assessed for their needs and linked to services they need, including for family planning.

“We can say it [family planning] is Integrated [with other PHC services]. First, any mother who visit the facility for different purposes like, OPD, nutrition will be counseled and linked to the family planning department and other Maternal and child health services. If she is pregnant, she will be linked to Antenatal care directly … during postnatal visit, she will be counseled and offered different types of family Planning services this is how we integrate family planning” Key informant, MCH focal person.

#### Management of inputs

Family planning is provided as one of the exempted services in Ethiopia. This has allowed users to access services free of user fees. This availability of “free” FP services provision is an opportunity for the integration of FP program with other PHC services. An informant pointed out this, “FP comes to us for free.” In-depth interviewee, client.

The integrated pharmaceuticals logistics system (IPLS) has created an opportunity to manage the family planning supply chain system to be run as part of the broader logistics system under the EPSA. According to a key informant from EPSA, this has created an opportunity to make sustainable changes in the supply of family planning commodities.

“That [integration of FP with other PHC services] is very good because it works well the integrated pharmaceutical logistics system. The items [family planning commodities] are distributed alongside with other medical items; so, it is important in providing items without any interruption, sustainability is the best benefit of this program.” Key informant, EPSA

#### Governance and financing

According to a key informant from an implementing partner/CSO, integration of FP with PHC is possible in Ethiopia, but it depends on the commitment of the leadership. As a result, the current level of integration varies from region to region with regions affected by the recent conflict having the lowest level of integration because of their competing priorities, like reconstruction of the health system.

Key informants indicated that there is a commitment from leadership at MOH and RHBs for FP integration with PHC. There exists FP-focused governance structures include the FP technical working group at the national level, which is composed of different stakeholders from the MOH and other partner organizations.

### Good practices

#### Service delivery

Post abortion and postpartum FP: Counseling services on FP now a days is part of a routine service integrated with ANC, postpartum services including post abortion care. A manager of a primary hospital described this by saying.

“When they [clients] come for abortion, if they had unwanted pregnancy, they will be counseled to use FP. Similarly, during postnatal care, they get counseled on postpartum FP … As they go hand in hand, we provide FP services during abortion and counseling on abortion during FP.” Hospital Manager.

Youth-friendly service centers: Youth-friendly service centers provide FP services integrated with other PHC services. They provide comprehensive maternal health care services which integrates FP as an important component of care provided at a one stop center. An interview with government official pointed out that:

“*Most of the health centers in our woreda have started providing youth-friendly services. As it provides special services to the youth, many youths have received FP services from youth-friendly service centers.*” Key informant, WoHO.

#### Management of inputs

Assigning professionals to liaise between MoH and EPSA to overcome challenges of the supply chain management system during the transition to IPLS helps to enhance supply of FP commodities.

The HEWs at the health posts provide limited FP services. Outreach sessions were used to overcome challenges related to lack of comprehensive FP options at health post level. An initiative in one of the study hospitals suggests that outreach from a higher level health facility to health posts may fill this gap by facilitating movement of trained personnel and FP commodities down to lower level health facilities. An interviewee from a primary hospital described outreach sessions as their good practice by saying:

“Our best practices are that … few months back we started providing mothers with long term FP services while the mothers were at their kebele health post. We send health care providers and give the service.” Key informant, Health Facility manager, Primary Hospital

#### Governance and financing

The importance of support provided by partners/development associations for FP service and integration was highlighted by a government official.

“Because we were supported by different NGOs such as IPAS, Amhara development association and Awi development Association …and we had strong review meetings quarterly, and best performing health professionals had got recognition from IPAS; this motivates them for better achievement.” Key informant, WoHO.

### Suggestions/recommendations to improve FP integration

Key stakeholders were also asked to give their suggestions on how to improve the current level of FP integration with other PHC services. The suggestions are summarized under service delivery, management of input and governance and financing and presented as follows.

#### Service delivery

It was suggested that the current FP service integration should be further strengthened with all maternal and child health services that are logically close to FP. Some suggested an integration model, where FP and other services are provided in one room over referral linkage where clients are referred from other departments for FP service. For this approach to be successful and sustainable, proponents of this approach recommended training of health workers on FP service provision so that they can provide integrated services.

On the other hand, other respondents recommended a pragmatic approach; i.e., full integration of FP in areas where full integration is possible. In this regard, it was suggested that FP should be fully integrated with at least some MCH services including, PNC, and CAC and clients should be able to get full package of FP services in one room.

In other areas, strengthening of the referral linkage in situations where full integration is not possible, because health workers may lack the necessary skills to provide FP services. For example, long-acting FP requires invasive surgical procedures and requires special skills. Another case is that quality of the service could be compromised if FP service is provided without proper counseling.

Some key informants believe that FP integration with other MCH services should be institutionalized to ensure sustainability of efforts, and it should happen at all levels of the health system, policy, program and service delivery points. Integration should happen in all areas such as planning, supply management and forecasting, and management information system.

#### Management of inputs

Shortage of FP commodities and skilled health workers are identified as the main challenges in FP integration. To address these challenges, respondents suggested a number of strategies, approaches and interventions including:

- Full integration of FP with other PHC services requires that all health professionals, not just the ones working in MCH, receive training on FP counseling and method provision. It is necessary to train all professionals on FP.
- Improving FP commodity supply chain through improving the supply chain management system at all levels of the health system, and strong coordination between the EPSA and health facilities.
- In the long term, allocation of adequate budget to FP commodities, to minimize the prevailing shortage of FP commodities, and to decrease donor dependency.
- It is also recommended that short-term training, mentorship, and supportive supervision of health workers improve their skills in providing good quality integrated FP services. The suggested topics for the training include FP counseling methods, long-acting FP provision, and FP commodities management system (forecasting, timely requesting and stock management). Online training approach was proposed to reach a wider audience with minimum resources and without disruption to the health service.
- Integration of FP with outreach child vaccination services, and mobile clinic services was also suggested for remote areas where FP service is inaccessible, particularly in pastoralist areas of Ethiopia. In addition, using Community Health Workers for health promotion and provision of some FP services in these settings are warranted.
- Provision of integrated FP service would increase the workload of health workers. To address this issue, devising incentive mechanisms including introducing performance-based payment scheme, including FP integration as a performance evaluation indicator were recommended. Moreover, using electronic MIS is recommended by some to integrate the information system and improve data quality.

#### Governance and financing

Key informants advocated engaging stakeholders including, schools and Women and Youth Affairs Offices in FP promotion and service provision. For instance, introducing FP and sexual education in school curriculums could improve awareness and coverage. The involvement of private health facilities in FP service provision is minimal. Most of FP services are currently provided by public health facilities. In this regard, engaging private service providers (private hospitals and private clinics) in FP service provision could reduce the burden of public health facilities and improve coverage.

Implementation of performance-based financing was mentioned as one of the motivation mechanisms for health care providers.

Despite improvements in recent years, lack of awareness and misconceptions about FP are still important barriers to FP use. In this regard, it was suggested to engage the community to increase awareness and dispel the prevailing misconceptions about FP. Involving religious leaders and community leaders as educators were repeatedly mentioned as effective strategies in addressing these misconceptions.

*“Giving training for influential people in the community on the benefits of FP using their own language and then making them to give training for the community in their towns would improve demand.”* Key informant, Health care provider

*“...It is important to teach the community that using FP is not prohibited by their religion and about the benefits of using FP method by involving the community and religious leaders.”* Key informant, *Woreda FP focal person*

Key informants discussed the importance of regular supply of FP logistics and of supplies to provide integrated FP service with good quality. Addressing budget shortage, gaps in coordination among organizations responsible for FP logistics, and gaps in forecasting FP needs (because of poor data quality, and weak logistics management system) were also discussed.

“As I just tried to explain to you, firstly enough manpower is needed to provide integrated FP services, secondly there must be enough supply, thirdly there must be enough space/room, these three things are very crucial. It would be difficult to say this without these being fulfilled. So, these must be fulfilled. I don’t think it’s difficult to do these three things in full.” Key informant, RHB

## Discussion

The current study explored the status of FP integration in Ethiopia, and opportunities, and challenges for integration. Our findings showed FP service is provided within PHC largely as a standalone service, and the state of integration with PHC service is limited and inconsistent. Some effective efforts by some health facilities include, integration of FP with abortion care, and Youth Friendly Service Centers. Facilitating factors for integration include, commitment of the government and partners to strengthen the integration of FP with PHC; presence of policies and guidelines that promote integration; evidence of successful integration of FP with abortion care; and positive attitude of managers, service providers, and beneficiaries on the idea of integration. However, our finding also revealed some major challenges that are largely related to challenges of the FP program itself. These include, resource shortage, health workers shortage, health workers capacity/skill gaps, and limited awareness and misconception of women, and cultural and religious norms.

Our finding identified a number of opportunities that facilitate successful integration of FP with PHC services. First, there is strong government commitment to promote FP integration with PHC service. National policy documents support the implementation of FP services as an integral component of other PHC services. There is commitment and ownership especially at the ministry of health level. The development of integration guidelines, inclusion of integration data elements in the health information system, and inclusion of FP logistics in the IPLS are evidence of integration of FP across the different components of the PHC system. These findings were also documented by previous studies that reported expansion of FP services through government led efforts in developing conducive policies, strategies and guidelines [29]. Second, high demand for FP by the community, presence of outreach programs for child vaccination, and presences of community health workers that provide FP are among the opportunities for implementing and further improving integrated FP service.

The Ethiopian government considered integration of FP with PHC services as a key strategy to reduce unmet need for FP. The recently revised FP guideline recommended integration of FP with a wide range of services including abortion care, maternal health services (ANC, delivery and postnatal care), child care services including immunization, HIV counseling, testing and care services, inpatient and outpatient services, and the model of integration can be either using an internal referral mechanism or direct provision of FP services depending on the context [24]. Though there are supportive environments and guidelines that promote effective FP integration, the level of integration was found to be inconsistent and vary from place to place and across the different components of the PHC system. Effective FP integration has happened with post-abortion care and Youth Friendly Service Centers. It appears, FP integration with abortion care is effective, because provision of FP is one component of comprehensive abortion care, as indicated in the Technical and Procedural Guideline for Safe Abortion Services in Ethiopia [30]. Previous studies also demonstrated that relatively large proportion of women were offered and used post abortion FP service [31, 32]. Whereas, integration of FP with other PHC services including MCH services appear limited, which lead to large proportion of, missed opportunity and unmet need for FP among women. In this regard, previous studies reported that large proportion of Ethiopian women still have unmet need for FP [13, 33]. The Ethiopian government strives to achieve the universal health coverage agenda by 2030 [29]. Expanding service integration, including FP integration could be one of the efficient strategies to achieve these agenda. Existing evidence consistently support the effectiveness of FP integration with other services in increasing use of FP and reducing unmet need for FP [15, 22].

The current study identified shortage of FP commodities as a challenge to the FP program, and in turn FP integration. It appears that the FP program is plagued by chronic shortage of FP commodities due to a number of reasons including, budget shortage, dry-up of donor support, delayed procurement, and problems related to supply chain management at all levels of the health system. To address these problems, the government has been broadening the resource base by increasing domestic financial resources allocated to the FP program to bridge the gap and decrease dependency of FP on external sources [29]. The newly rolled out Community Based Insurance scheme is expected to improve the current budget gap of the government health system, although FP service is provided free of charge [34].

Studies in southwest Ethiopia stated issues related to shortage of medical equipment and supplies, trained staffs, and information education and communication materials (IEC) as main explanations for the low quality of FP service and integration [35]. Shortage of drugs and supplies including HMIS supplies were found important challenges. A study conducted in Kenya showed improving logistics supply increased Family planning utilization [36]. This study also indicated that critical steps need to be taken in resolving drug and supply problems in general and long-term FP contraceptive methods in particular. On the other hand, the distance of the health facility from the supply's distribution centers and lack of supportive supervision needs to be considered important. Studies in Zambia revealed lack of transportation was among the challenges that must be considered as obstacles for FP movement particularly in rural and border areas [36].

In order to achieve effective FP integration with PHC services, there needs to be sufficient number of health workers that have the required skill to provide FP services in addition to what is required of them or refer clients to FP. In most cases, in-service training to health workers could be required to improve health workers knowledge and skills [37]. In this regard our study disclosed the shortage of health workers and lack of skill of available health workers as an obstacle for effective integration of FP services. Frequent turnover and high workload of service providers were also identified as major challenges to integrate FP with PHC services. Previous studies from different contexts also reported similar challenges [38, 39]. Providing vehicles, regular supervision and support to the health facilities help to improve the capacity of service delivery.

Family planning services, particularly long-term FP methods were not provided at the health posts because of unavailability of qualified health professionals at the health post level. The services are being provided at the health centers and hospitals. However, this study found that there are health centers that started providing outreach services for mothers in their villages. This type of approaches can enable women access the services easily at their village. This can be taken as the best experience and shared with others. Engagement of community and religious leaders to deliver health education to the community including integrated PHC –FP program was also indicated as a good approach for better performance of the program implantation.

Our finding also revealed that overall awareness of women about FP and demand for FP is increasing over time. However, in remote and pastoralist areas, awareness level is low, and demand side barriers for FP use are major challenges. For instance, religious and cultural norms, limited awareness and misconceptions of women about FP are found to be major challenges, indicating the need to address prevailing structural challenges to improve coverage and promote equity. In fact, the national FP guideline acknowledge the significant inequity in FP use, and recommended social and behavioral change communication, and community level interventions [24]. Gender sensitive and culturally sensitive intervention to address demand side barriers is recommended by some studies [40].

Some studies from Ethiopia and other African countries reported that there are perceptions among communities that contraceptives could induce illness; FP is considered a sin; lack of acceptance by community leaders including religious leaders, unwillingness and disagreements from husbands were among the barriers for implementation of integrated FP services. In these studies, participants discussed the role of societal perception, myths-misunderstanding and husband/male engagement as barriers to access and integrate FP services. Increasing population awareness on fertility related problems and providing all sorts of support aimed at integrated PHC—FP will help to better implementation of integrated PHC-FP service delivery [41–44].

The current study should be read in light of the following strengths and limitations. The study is comprehensive in that it included views of diverse FP stakeholders and experts representing different organizations including the government, partners supporting the FP program, service providers and clients. Proper data quality control mechanisms were in place during data collection, management and analysis. However, the study focused only on FP integration efforts of public health facilities, experiences of private-for-profit health facilities, and health facilities run by non-governmental organizations are not addressed. Since our study is purely qualitative, and study participants are selected purposively, the finding may lack representativeness, and generalizability.

## Conclusions

Integration of FP with PHC services in the Ethiopian public health facilities is viable. Government commitment, and partners’ support, presence of policy documents promoting integration and anecdotal evidence of successful integration in some facilities are facilitators for integration. However, pre-existing challenges of the FP program, including resource shortage, health workers shortage and limited capacity and some religious and cultural norms are barriers to integration. Expanding the experiences of good practices in the integration of FP with post abortion care, post-natal care, HIV care, and youth-friendly service centers to other components of PHC warrants attention. Addressing supply side problems through in-service training of health workers, joint planning and management to improve efficiency is needed. Structural barriers such as misconception and cultural norms should be addressed through culturally-sensitive behavior change communication, community mobilization and dissemination of health information.

## Data Availability

Transcripts from all key informant interviews are stored at the data center of EngenderHealth-Ethiopia. They can be accessed through correspondence with the corresponding author upon reasonable request.

## Abbreviations

EPSA: Ethiopian pharmaceutical supplies agency
FP: Family planning
LMICs: Low- and middle-income countries
MCH: Maternal and child health
MOH: Ministry of health
PHC: Primary health care
SDG: Sustainable development goals
WHO: World Health Organization
WoHO: Woreda health office
